# Soluble Urokinase Plasminogen Activator Receptor (suPAR) Independently Predicts Severity and Length of Hospitalisation in Patients With COVID-19

**DOI:** 10.3389/fmed.2021.791716

**Published:** 2021-12-02

**Authors:** Helena Enocsson, Cornelia Idoff, Annette Gustafsson, Melissa Govender, Francis Hopkins, Marie Larsson, Åsa Nilsdotter-Augustinsson, Johanna Sjöwall

**Affiliations:** ^1^Division of Inflammation and Infection, Department of Biomedical and Clinical Sciences, Linköping University, Linköping, Sweden; ^2^Department of Infectious Diseases, The Vrinnevi Hospital, Norrköping, Sweden; ^3^Division of Molecular Medicine and Virology, Department of Biomedical and Clinical Sciences, Linköping University, Linköping, Sweden

**Keywords:** suPAR, COVID-19, biomarker, disease severity, respiratory failure, length of hospital stay

## Abstract

**Background:** Efficient healthcare based on prognostic variables in hospitalised patients with COVID-19 could reduce the risk of complications and death. Recently, soluble urokinase Plasminogen Activator Receptor (suPAR) was shown to predict respiratory failure, kidney injury, and clinical outcome in patients with SARS-CoV-2 infection. The aim of this study was to investigate the value of suPAR as a prognostic tool, in comparison with other variables, regarding disease severity and length of hospital stay in patients with COVID-19.

**Patients and Methods:** Individuals hospitalised with COVID-19 (40 males, 20 females; median age 57.5 years) with a median symptom duration of 10 days and matched, healthy controls (*n* = 30) were included. Admission levels of suPAR were measured in serum by enzyme-linked immunosorbent assay. Blood cell counts, C-reactive protein (CRP) levels, lactate dehydrogenase (LDH), plasma creatinine and estimated glomerular filtration rates were analysed and oxygen demand, level of care and length of hospitalisation recorded.

**Results:** Patients had significantly higher suPAR levels compared to controls (*P* < 0.001). Levels were higher in severely/critically (median 6.6 ng/mL) compared with moderately ill patients (median 5.0 ng/mL; *P* = 0.002). In addition, suPAR levels correlated with length of hospitalisation (rho = 0.35; *P* = 0.006). Besides suPAR, LDH, CRP, neutrophil count, neutrophil-to-monocyte and neutrophil-to-lymphocyte ratio, body mass index and chronic renal failure were discriminators of COVID-19 severity and/or predictors of length of hospitalisation.

**Conclusion:** Admission levels of suPAR were higher in patients who developed severe/critical COVID-19 and associated with length of hospital stay. In addition, we showed that suPAR functioned as an independent predictor of COVID-19 disease severity.

## Introduction

The ongoing pandemic caused by the novel severe acute respiratory syndrome coronavirus 2 (SARS-CoV-2) has caused extensive morbidity and deaths, which has entailed an enormous burden on the healthcare system worldwide. The SARS-CoV-2 can cause an asymptomatic to severe coronavirus disease 2019 (COVID-19), and when severe has serious impact primarily on the lungs, but also on several other organs (1–3). Pneumonia with subsequent respiratory failure may develop already within the first week after disease onset (4–6), leading to hospitalisation of patients in need of oxygen supplementation or with multiple organ dysfunction. At this stage of the infection, it is difficult to predict which patients will need intensified oxygen supplementation, either as high-flow nasal oxygen therapy (HFNOT), continuous positive airway pressure (CPAP) therapy, or mechanical ventilation, and to estimate the length of hospital stay. Prognostic markers are essential for identifying patients at risk of developing severe COVID-19, so that appropriate care interventions can be offered prophylactically or at least at an early stage of the disease. Certain blood-based biomarkers, such as lymphocyte, neutrophil and platelet counts, neutrophil-to-monocyte ratio (NMR), neutrophil-to-lymphocyte ratio (NLR), D-dimer, interleukin-6, C-reactive protein (CRP) levels and lactate dehydrogenase (LDH) may discriminate between severe and non-severe COVID-19 (4, 7, 8). Another biomarker, the soluble urokinase plasminogen activator receptor (suPAR), has been shown to be significantly elevated in patients with COVID-19 (9), and stands out as a predictor of overall disease severity and outcome (10–15) and in particular of severe respiratory failure (16), and acute kidney injury (17) due to SARS-CoV-2 infection.

The cell-bound urokinase-type plasminogen activator receptor (uPAR) is found on various cell types such as endothelial cells and activated neutrophils and is upregulated at sites of inflammation and tissue remodelling (18, 19). Apart from urokinase plasminogen activator (uPA, also known as urokinase), uPAR interacts and cooperates with many ligands and receptors, primarily integrins, to facilitate intracellular signalling, cell migration, cell adhesion and tissue remodelling (20). Soluble uPAR (suPAR) results from proteolytic cleavage of uPAR (21), and has gained increased interest as circulating levels are found to reflect severity and prognosticate outcome of several malignant (22–26), autoimmune (27–30) and infectious diseases (31–37). Additionally, suPAR is used in triaging of patients in acute care settings to early predict clinical deterioration due to suspected bacterial infections (38–42).

As suPAR has only recently been suggested to predict the outcome of COVID-19, and its significance in hospitalised patients with different disease severity is not yet fully understood, we conducted this prospective cohort study with the aim to evaluate whether suPAR, in comparison to established blood-based immune mediators, may prognosticate respiratory failure and length of hospital stay in adult patients with SARS-CoV-2 infection.

## Materials and Methods

### Study Design and Participants

This study was part of a prospective, observational cohort study, implemented during August 2020 to May 2021, involving consecutive adult patients with COVID-19, who were assessed for their eligibility of inclusion as soon as possible following admittance to the Department of Infectious Diseases at the Vrinnevi Hospital, Norrköping, Sweden. Inclusion criteria were age >18 years, ability to give informed consent and with a current diagnosis of COVID-19, as verified by Abbott Real Time SARS-CoV-2 or Alinity m SARS-CoV-2 assays (Abbott, Solna, Sweden) using nasopharyngeal or throat swab specimens, performed at the Clinical Microbiology Laboratory, Linköping University Hospital, Sweden.

Healthy, SARS-CoV-2 RNA negative controls, verified by an in-house RealTime quantitative PCR performed as previously described (43), were matched to the patients regarding age and sex and were only used for comparison of suPAR levels. They consisted of health care workers at the Vrinnevi Hospital (*n* = 17) and blood donors (*n* = 13) at the Department of Clinical Immunology and Transfusion medicine, Linköping University Hospital, Sweden.

### Clinical Characteristics of Patients, Disease Severity Classification and Biochemical Variables

At inclusion, patients were asked by a questionnaire about the duration of COVID-19-associated symptoms and smoking habits. Digital medical records were reviewed with respect to various factors: presence of cardiovascular disease (CVD, including hypertension), chronic pulmonary disease (asthma, chronic obstructive pulmonary disease, pulmonary fibrosis or other chronic illnesses affecting the lungs), chronic renal failure (CRF), diabetes, current medication, date of confirmed SARS-CoV-2 infection, immunosuppression (disease and/or current medical treatment that suppress the immune system), length of hospital stay, highest level of care received, maximum need of oxygen supplementation (<5 L/min or HFNOT/CPAP/mechanical ventilation), need of renal dialysis, and lastly, COVID-19 related medication (anticoagulants, remdesivir and/or dexamethasone).

In the patient cohort of hospitalised patients, COVID-19 severity was classified according to the National Institute of Health (44) and approximated with respect to the highest level of care (pandemic department, intermediate or intensive care unit) and the maximum oxygen need as *mild* (pandemic department, no oxygen supplementation), *moderate* (pandemic department, oxygen supplementation <5 L/min), *severe* (pandemic department or intermediate care unit, oxygen need ≥5 L/min supplemented by HFNOT or CPAP) and *critical illness* (intensive care unit, with or without mechanical ventilation). Because only a few patients were classified as mild and critical, respectively, two groups of disease severity (mild/moderate and severe/critical) were used in analyses throughout this study.

Patients were analysed for baseline haemoglobin, blood cell counts (platelets, leukocytes, monocytes, lymphocytes, neutrophils, eosinophils, and basophils), CRP, LDH, sodium, potassium and plasma creatinine at the Clinical Chemistry Unit, Vrinnevi Hospital, Norrköping. The estimated glomerular filtration rate (eGFR) was calculated using the MDRD 4-variable equation (45). An eGFR of >90 mL/min/1,73 m^2^ was not further specified and was given the value of 100 mL/min/1,73 m^2^. Body mass index (BMI), the neutrophil-to-monocyte (NMR) and neutrophil-to-lymphocyte ratios (NLR) were calculated.

### SuPAR Analysis

Serum from patients and controls was prepared from venous whole blood, drawn at study inclusion, and stored at −80°C until analysis. Serum suPAR was measured in duplicates by the clinically validated suPARnostic ELISA kit (Virogates, Birkerød, Denmark) according to the manufacturer's instructions. In brief, samples, standards and controls were mixed with peroxidase conjugated anti-suPAR antibodies and thereafter transferred to a 96-well plate, precoated with anti-suPAR antibodies. After 1 h of incubation in room temperature and subsequent washing of the plate, 3',3',5',5'-tetramethylbenzidine was added. The reaction was stopped after 20 min of incubation by the addition of sulphuric acid, and the absorbance was measured at 450 nm with 650 nm as reference wavelength, in an absorbance reader (SpectraMax ABS Plus from Molecular Devices, LCC, San Jose, CA, USA) and analysed using Softmax Pro 7 (Molecular Devices, LCC). The curve controls, accompanied with the ELISA kit, were within range in all assays. Serum samples with optical density values above the range of the standard curve were diluted 1:5 and re-run. The mean coefficient of variation between duplicates was 6.1% (range 0.1–25.8%).

### Statistical Analysis

suPAR was not normally distributed and hence, non-parametric tests were generally used. Mann-Whitney *U*-test was always used when two groups were compared, and Spearman's correlation was used for all correlation analyses. χ^2^ test with Fisher's exact method was used for categorical data. Kruskal Wallis with Dunn's multiple comparison was used when more than two groups were compared. For linear regression analysis, the dependent variable (length of hospital stay) was log-transformed prior to analysis to achieve a normal distribution. Values below limit of quantitation were given half the value of the limit. A two-sided *P*-value ≤ 0.05 was considered significant. IBM SPSS Statistics, version 23 was used for statistical calculations. GraphPad Prism 9.1.2 software (GraphPad, La Jolla, CA, USA) was used for the graphical illustrations.

### Ethical Considerations

Oral and written informed consent was obtained from all participants. The study protocol was approved by the Swedish Ethical Review Authority (Decision number 2020–02580).

## Results

### Characteristics of Patients and Controls

Sixty hospitalised patients (40 males [67%], median age 57.5 years [range 23–91]) with confirmed COVID-19 constituted the patient cohort. The median age among the controls was 56 years (range 31–69) and 66% (*n* = 20) were males. Five individuals were above 75 years of age, as the very old patients to a large extent had confusion and or dementia or were unable to participate for other reasons.

Fifty-seven percent (*n* = 34) were ever smokers and 25% (*n* = 15) had chronic pulmonary disease. CVD, including hypertension, was present in 57% (*n* = 34) of the patients, diabetes in 25% (*n* = 15) and the median BMI was 30 (range 22–45). Seven (12%) patients had CRF, whereof one received intermittent haemodialysis (Table 1). Acute renal failure (including those with acute on CRF) was observed in ten (17%) patients at inclusion, and two needed continuous renal replacement therapy at the intensive care unit (Table 1). Eight (13%) patients were considered immunocompromised, whereof one had acute myeloid leukaemia and another two multiple myeloma receiving cytostatic treatment, respectively, one had newly diagnosed chronic lymphatic leukaemia without active treatment, two had undergone kidney transplantation and were treated with cyclosporine and mycophenolate mofetil and two had spondyloarthritis receiving treatment with tumour necrosis factor inhibitors (adalimumab and etanercept, respectively) (Table 1). The median symptom duration at inclusion was 10 days (range 2–30) and the median length of hospital stay one week (2–54 days). The length of hospital stay was considered a surrogate measure of disease severity, and since the maximum length of stay in discharged patients was 54 days, the deceased patients were given a fictive stay of 55 days to avoid bias. All patients received, according to current recommendations, anticoagulant therapy (low molecular weight heparin or continued with direct acting oral anticoagulants or warfarin) at admission to hospital. At study inclusion, 23 (38%) patients received or had previously received the antiviral treatment remdesivir (200 mg day 1, followed by 100 mg once daily for 5–10 days) and 38 (63%) were prescribed either oral or intravenous dexamethasone (6 mg once daily for up to 10 days), betamethasone (4 mg once daily for 5 days) or prednisolone (30 mg per day for 5 days) (Table 1).

**Table 1:** Clinical characteristics of and biochemical variables in the patient cohort.

**Patients, number (*n*) 60**	
Male sex, *n*, (%)	40 (67)
Age, years median (range)	57.5 (23–91)
Current or ex-smoker, *n* (%)	34 (57)
Cardiovascular disease, *n* (%)	34 (57)
Chronic pulmonary disease, *n* (%)	15 (25)
Acute renal failure, *n* (%)	10 (17)
Chronic renal failure, *n* (%)	7 (12)
Renal replacement therapy, *n* (%)	3 (5)
Diabetes, *n* (%)	15 (25)
Body mass index (kg/m^2^), median (range)	30 (22–45)
Immunocompromised^#^ at inclusion, *n* (%)	8 (13)
Symptom duration, days median (range)	10 (2–30)
Length of hospital stay, days median (range)	7 (2–54)
Deceased, *n* (%)	2 (3)
**COVID-19 severity:**	
Mild, *n* (%)	6 (10)
Moderate	25 (42)
Severe, *n* (%)	21 (35)
Critical, *n* (%)	8 (13)
Mechanical ventilation, *n* (%)	4 (7)
Mechanical ventilation, days median (range)	13.5 (2–21)
Intensive care, *n* (%)	8 (13)
Stay at intensive care unit, days median (range)	9 (1–24)
Anticoagulants, *n* (%)	60 (100)
Remdesivir, *n* (%)	23 (38)
Corticosteroid therapy, *n* (%)	38 (63)
**Biochemical variables at inclusion:**	
Haemoglobin g/L, median (range)	127 (87–171)
White blood cell count (x 10^9^/L), median (range)	6.7 (0.4–47)
Platelet count (x 10^9^/L), median (range)	240 (20–668)
Neutrophil count (x 10^9^/L), median (range)	5.0 (0.1–17.5)
Lymphocyte count (x 10^9^/L), median (range)	1.0 (0.1–33)
NLR, median (range)	4.9 (0.2–88)
NMR, median (range)	13.1 (4.0–180)
C-reactive protein (mg/L), median (range)	62.5 (6–477)
Sodium (mmol/L), median (range)	138 (128–144)
Potassium (mmol/L), median (range)	3.9 (3.2–4.8)
Plasma creatinine (μmol/L), median (range)	69.5 (36–1224)
eGFR MDRD (mL/min/1.73m^2^), median (range)	69 (4–>90)
Lactate dehydrogenase (μkat/L), median (range)	6.5 (3.2–16)

*# Disease and/or current medical treatment that suppress the immune system, such as haematological or other malignancy, rheumatic disease, and organ transplantation. HFNOT, high flow nasal oxygen therapy; eGFR, estimated glomerular filtration rate; NLR, neutrophil-to-lymphocyte ratio; NMR, neutrophil-to-lymphocyte ratio. Disease severity was classified according to the National Institute of Health and approximated with respect to maximum oxygen need as mild (pandemic department, no oxygen supplementation), moderate (pandemic department, oxygen supplementation <5L/min), severe (pandemic department or intermediate care unit, oxygen need ≥5L/min supplemented by high-flow nasal oxygen therapy or continuous positive airway pressure therapy) and critical illness (intensive care unit, with or without mechanical ventilation)*.

Baseline blood cell counts, CRP levels, LDH, sodium, potassium, plasma creatinine, eGFR, BMI, NMR and NLR in the patient cohort are presented in Table 1.

### Classification of COVID-19 Severity

COVID-19 severity was classified as *mild* in six (10%), *moderate* in 25 (42%), *severe* in 21 (35%) and *critical* in 8 patients (13%), of whom all eight needed intensive care (seven males, one female; median length of stay 9 days [1–24]), whereof four (7%) males received mechanical ventilation (median 13.5 days, range 2–21). One of the critically ill, mechanically ventilated patients had a kidney transplant. Two (3%) male patients needed continuous renal replacement therapy at the intensive care unit. Two male patients deceased during the hospital stay, whereof one had acute myeloid leukaemia and the other had CVD, obesity, and diabetes. The former patient was treated at an intermediate care unit (8 days) and the other received intensive care (22 days) with mechanical ventilation and continuous renal replacement therapy.

### SuPAR in Relation to Patient Characteristics and Biochemical Variables

**Figure 1:**
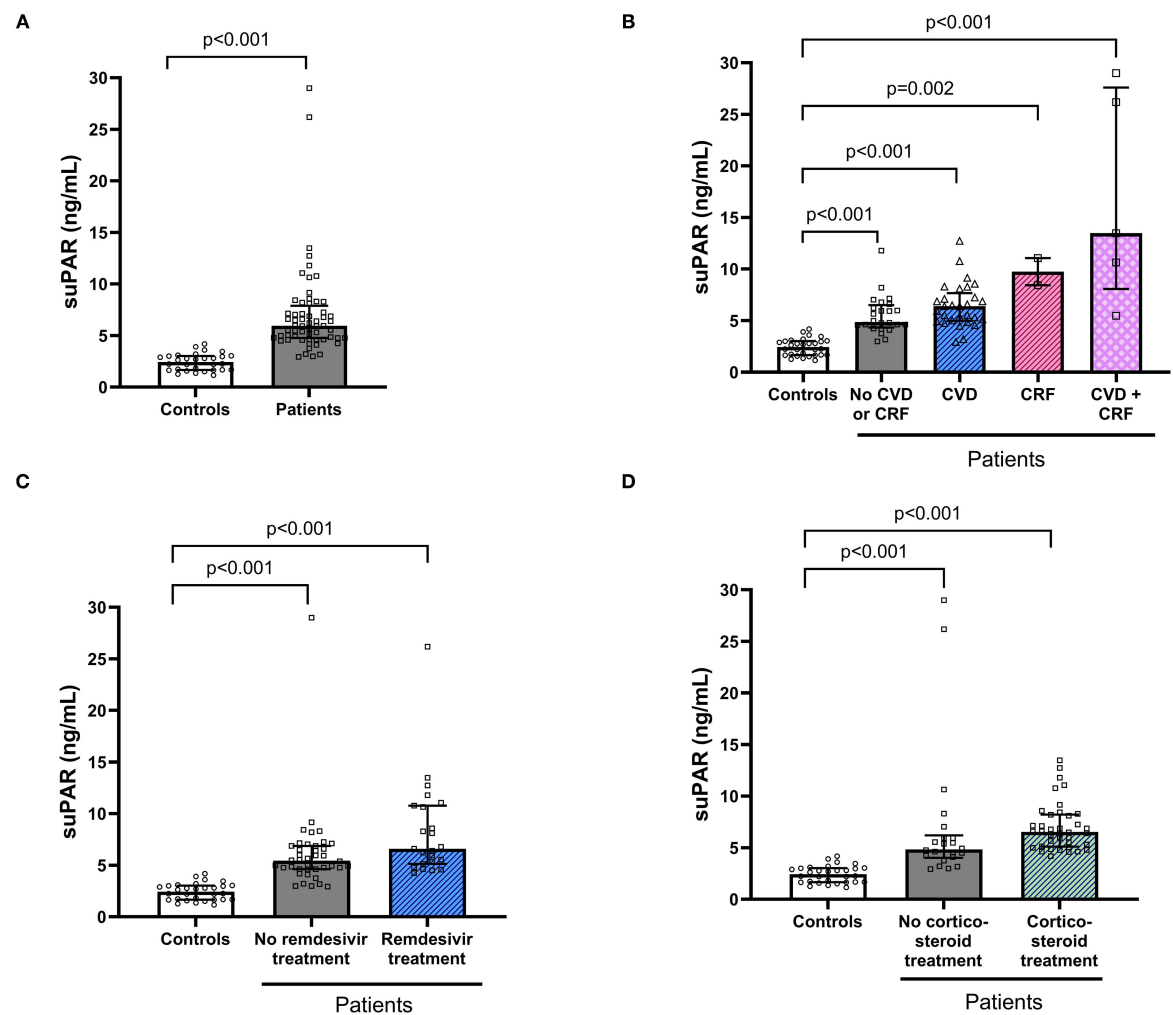
Soluble urokinase plasminogen activator receptor (suPAR) levels in controls and patients hospitalised with COVID-19. suPAR serum concentration was compared between individuals hospitalised with COVID-19 and healthy age- and sex-matched controls **(A)**. COVID-19 patients were also stratified based on comorbidities **(B)** and treatments **(C,D)** of importance for suPAR levels and thereafter compared with the control group. Bars and error bars show median and inter quartile range. Dots represent individual values. suPAR, soluble urokinase plasminogen activator receptor; CVD, cardiovascular disease; CRF, chronic renal failure.

The COVID-19 patients had significantly higher baseline suPAR levels (median 5.9, IQR 4.8–7.9) compared to the matched controls (median 2.44, IQR 1.66–3.02, *P* < 0.001; Figure 1A). Among patients, suPAR was correlated with age (*P* > 0.001, rho = 0.42) and length of hospital stay (*P* = 0.006, rho = 0.35) but not with BMI, or symptom duration. Significantly higher suPAR levels were found among patients with CRF (*P* < 0.001) and CVD (*P* = 0.043) compared to patients without the respective disorders. Furthermore, patients treated with remdesivir had higher suPAR levels (*P* = 0.03) compared to patients without remdesivir. Higher suPAR levels were also found among patients with corticosteroid treatment (*P* = 0.006). For this reason, patients were also stratified based on CVD and/or CRF (Figure 1B), remdesivir treatment (Figure 1C) and corticosteroid treatment (Figure 1D) for comparison with the healthy controls. This analysis revealed that all patient subgroups had significantly higher suPAR levels in comparison with the healthy control group (Figures 1B–D).

No statistically significant differences in suPAR levels were found among the patients considering biological sex, smoking habits, immunosuppressive disorders, presence of chronic pulmonary disease, or diabetes.

Biochemical factors that correlated significantly with suPAR levels were plasma creatinine (*P* = 0.008, rho = 0.34) and eGFR (*P* = 0.012, rho = −0.32). No correlation was found with CRP levels, LDH, haemoglobin, blood cell counts, NMR, NLR, or electrolytes.

### SuPAR, and Other Biochemical and Clinical Variables in Association With Length of Hospital Stay

**Figure 2:**
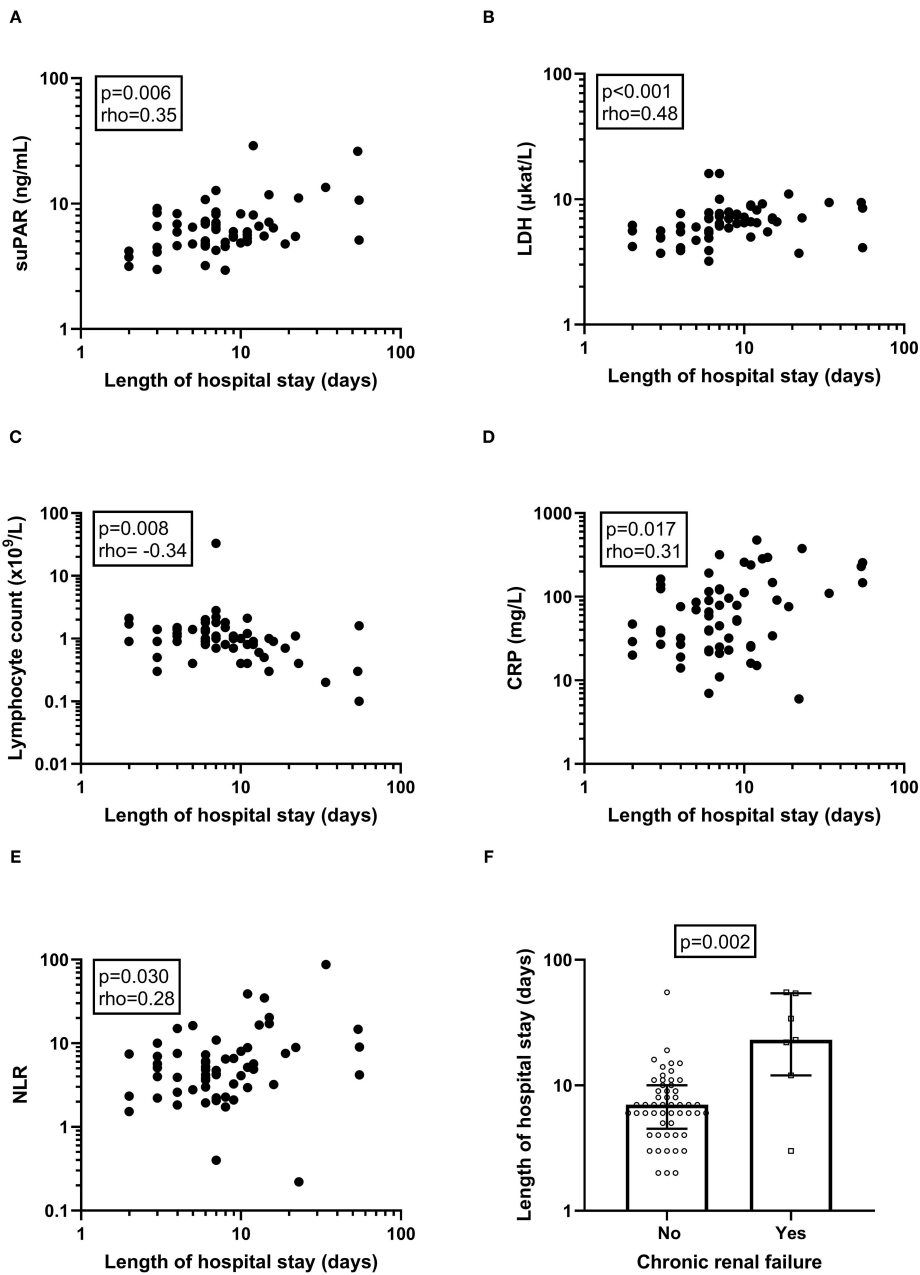
Correlations and associations of biochemical and clinical variables with length of hospital stay. The correlation between the length of the hospital stay and biochemical **(A–E)** and clinical variables **(F)** were examined. Deceased patients (*n* = 2) were given a fictive length of stay of 55 days to avoid bias. *P*-value and Spearman's correlation coefficient (rho) is given for each correlation analysis. *P*-value from Mann-Whitney *U*-test is given for CFR. Please observe that axes display a logarithmic scale. suPAR, soluble urokinase plasminogen activator receptor; LDH, lactate dehydrogenase; CRP, C-reactive protein; NLR, neutrophil-to-lymphocyte ratio.

The length of hospitalisation significantly associated with suPAR levels (*P* = 0.006, rho = 0.35), LDH (*P* < 0.001, rho = 0.55), lymphocyte count (*P* = 0.008, rho =−0.34), CRP (*P* = 0.017, rho = 0.31) and NLR (*P* = 0.030, rho = 0.28) (Figures 2A–E), respectively. No correlation was found between length of hospital stay and other immune cell counts, sodium, potassium, plasma creatinine, or eGFR (not shown). Among the clinical variables, only CRF was significantly associated with length of hospital stay (*P* = 0.002; Figure 2F). Remdesivir treated patients had significantly longer hospital stay (median 8 days, range 3–54, *P* = 0.004), whereas no significant difference in length of hospital stay was found dependent on corticosteroid treatment.

### Predictors of Length of Hospital Stay in a Multiple Linear Regression

A linear regression model with stepwise analysis revealed that none of the potential confounders (age, eGFR, corticosteroid and remdesivir treatment) abolished the association of suPAR with length of hospital stay (*P* = 0.004). Only remdesivir treatment remained significant (*P* = 0.007) in the regression model together with suPAR (not shown).

To create an optimised model for prediction of length of hospital stay, all biochemical variables (LDH, suPAR, lymphocyte count, CRP and NLR) that were significantly associated with this outcome variable were attested by linear regression. In the analysis including the lymphocyte count (Table 2, Model 1), only suPAR (*P* = 0.001) remained an independent variable, whereas LDH, lymphocyte count and CRP were excluded from the model (Table 2). A linear regression model with stepwise analysis of suPAR, NLR, LDH and CRP proved suPAR (*P* = 0.003) and NLR (*P* = 0.022) to be significantly associated with length of hospital stay, while LDH and CRP were excluded from the model (Table 2, Model 2).

**Table 2:** Biochemical variables associated with length of hospital stay (10log) in stepwise linear regression analyses.

	***P*-value**	**Standardised beta**	**Adjusted model R^**2**^**
**Model 1**			0.16
suPAR	0.001	0.42	
*Excluded from the model:*			
Lactate dehydrogenase			
Lymphocyte count^*^			
CRP			
**Model 2**			0.23
suPAR	0.003	0.37	
NLR^*^	0.022	0.28	
*Excluded from the model:*			
Lactate dehydrogenase			
CRP			

*suPAR; soluble urokinase activator receptor, NLR; neutrophil-to-lymphocyte ratio, CRP; C-reactive protein*.

* *Lymphocyte count and NLR were tested in separate models because of their interrelationship*.

Among clinical variables, CRF and remdesivir treatment had a significant positive correlation with length of hospital stay. A linear regression model including suPAR, CFR and remdesivir abolished the association of suPAR with length of hospitalisation. Although none of the other clinical variables were significantly associated with suPAR, age, BMI and male sex are known risk factors of hospitalisation and development of severe COVID-19 (46). Hence, these three variables were tested together with suPAR and NLR but did not remain significant in the stepwise analysis.

COVID-19 severity was also tested together with suPAR to reveal if the association of suPAR and length of hospital stay was dependent on the disease severity. This linear regression analysis revealed that both COVID-19 severity (*P* = 0.002) and suPAR levels (*P* = 0.013) are positively associated with the length of hospital stay.

### SuPAR and Its Association With COVID-19 Severity

**Figure 3:**
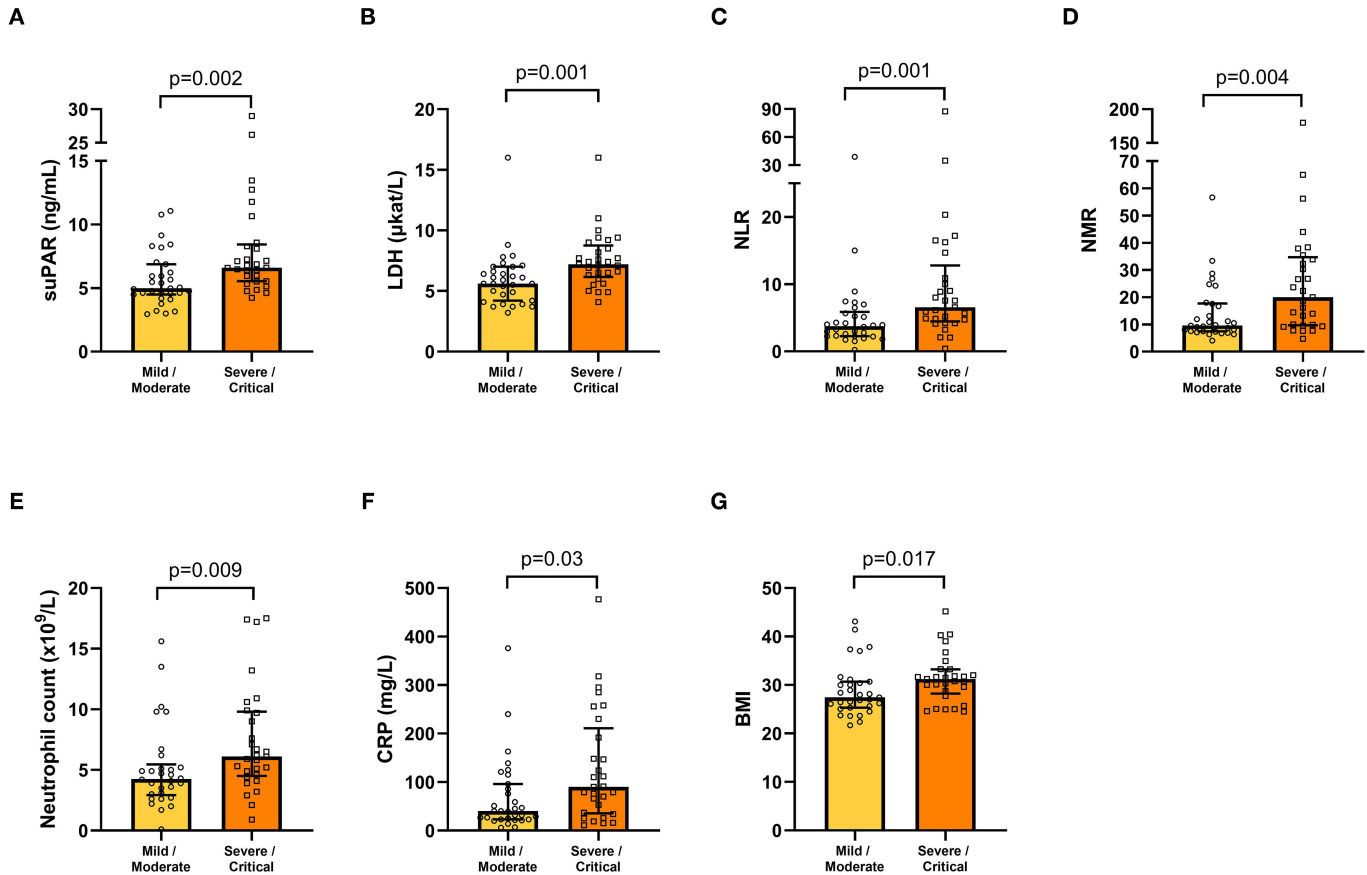
Biochemical **(A–F)** and clinical (BMI; **G**) variables, which are significantly increased in COVID-19 patients with severe/critical compared with mild/moderate illness. Bars and error bars represent median values and inter quartile range, respectively. Circles and squares represent individual values. BMI, body mass index; CRP, C-reactive protein; LDH, lactate dehydrogenase; NLR, neutrophil-to-lymphocyte ratio; NMR, neutrophil-to-monocyte ratio; suPAR, soluble urokinase plasminogen activator receptor.

The association of suPAR and other biochemical and clinical variables with COVID-19 severity was tested by comparison of the mild/moderate disease group of patients (*n* = 31) with the group of severely/critically ill patients (*n* = 29). suPAR, LDH, NLR, NMR, neutrophil count, and CRP were found to be significantly increased in the severe/critical group of patients, compared with the mild/moderate group (Figures 3A–F). Among clinical variables, only BMI was significantly different between the two severity groups (Figure 3G). Age, symptom duration, haemoglobin, cell counts (platelets, leukocytes, lymphocytes, monocytes, eosinophils, basophils), sodium, potassium, plasma creatinine and eGFR were not significantly different between the two severity groups (not shown).

χ^2^ tests for binary variables (sex, current/previous smoking status, presence of CVD, chronic pulmonary disease, diabetes, CRF or remdesivir treatment) in relation to COVID-19 severity did not reveal any statistically significant associations. Corticosteroid treated patients were significantly overrepresented in the severe/critical group of patients (*P* = 0.003, not shown).

### Predictors of COVID-19 Severity Evaluated by a Logistic Regression Analysis

**Figure 4:**
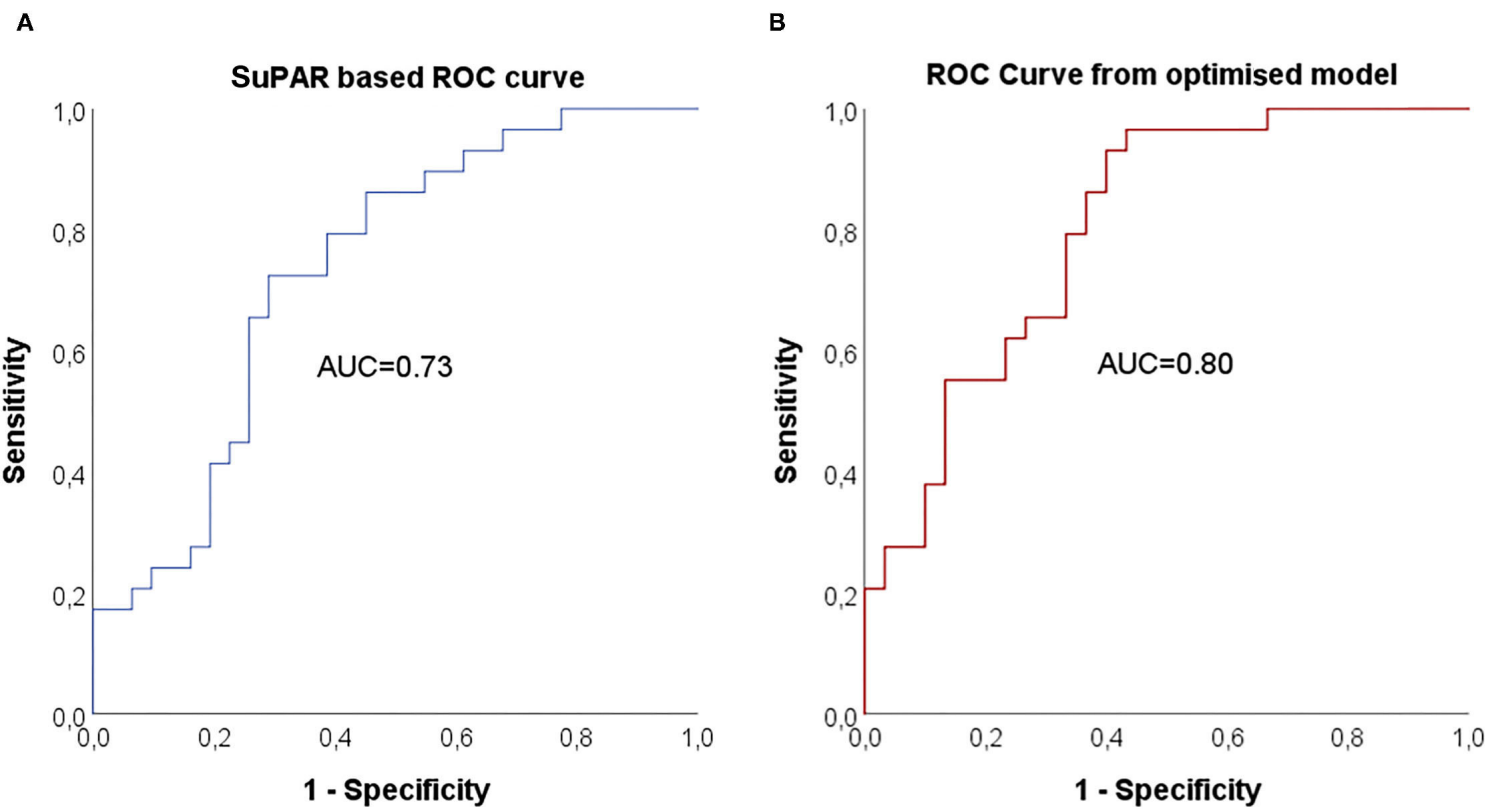
Receiver operating characteristic (ROC) curve and area under curve (AUC) for prediction of COVID-19 severity. AUC for suPAR based prediction of COVID-19 severity **(A)** and AUC for predicted probabilities from the optimised model with suPAR, BMI and NLR **(B)**.

Baseline variables associated with COVID-19 severity were combined in a stepwise logistic regression model (forward: conditional) to evaluate suPAR in relation to the other variables (Table 3). Due to the interrelationship between neutrophil count, NLR and NMR, only NLR was tested together with suPAR, LDH, CRP and BMI. suPAR levels or predicted probabilities from the optimised regression model were thereafter used to create receiver operator characteristics (ROC) curves for each model, where AUC was calculated (Table 3, Figure 4). An optimal suPAR cut-off at 5.9 ng/mL was achieved at a specificity of 71% and a sensitivity of 72%.

**Table 3:** Binary logistic regressions for the outcome of severe/critical COVID-19 disease (versus mild/moderate disease).

**Model**	**AUC (95% CI)**	**Baseline variable**	**OR (95% CI)**	***P*-value**
Only suPAR	0.73 (0.61–0.86)	suPAR	1.35 (1.04–1.75)	0.023
Optimised	0.80 (0.69–0.91)	suPAR	1.42 (1.04–1.94)	0.025
		BMI	1.16 (1.02–1.33)	0.024
		NLR	1.11 (0.99–1.23)	0.077
		LDH (excluded)	N/A	N/A

### SuPAR Based Stratification in Relation to COVID-19 Severity

**Figure 5:**
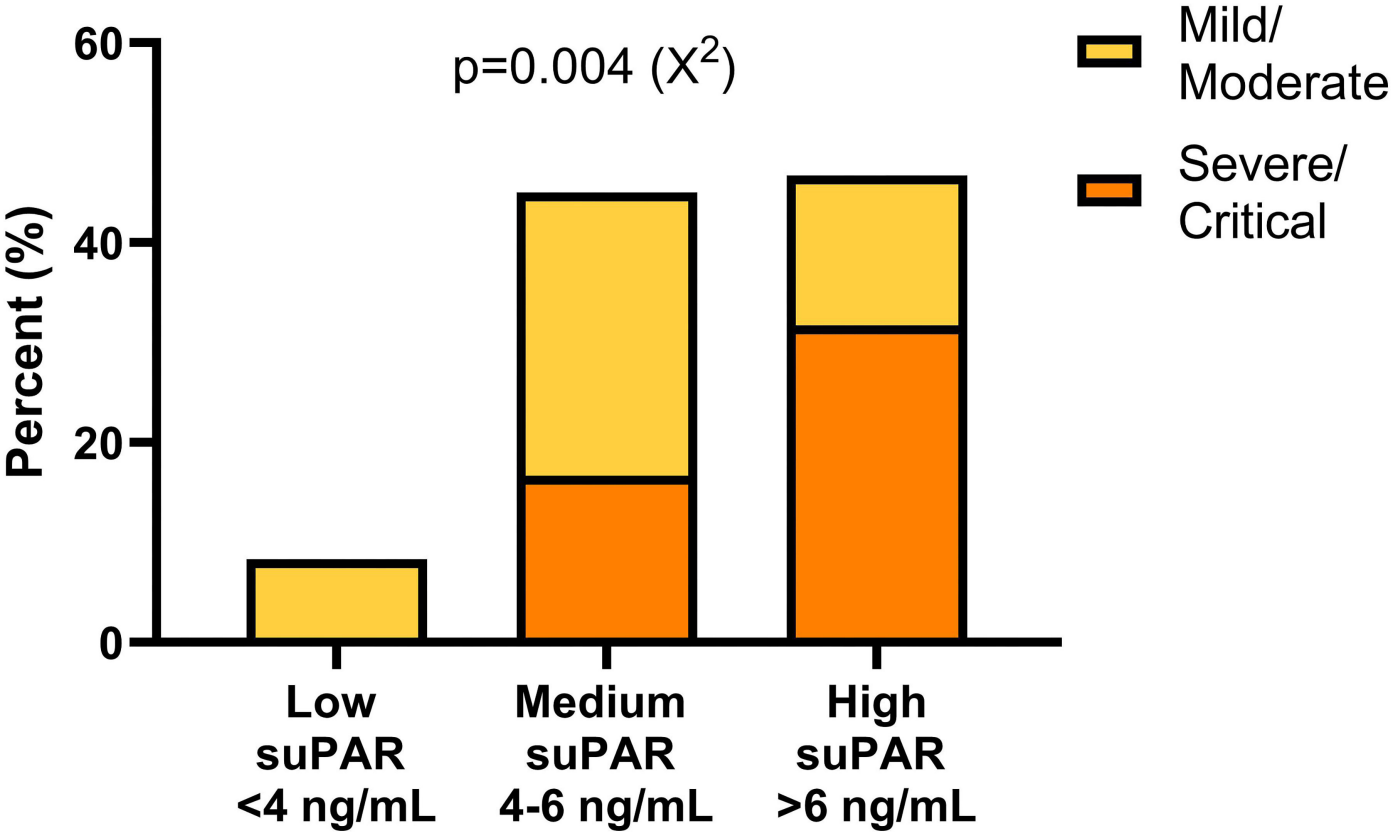
Frequencies of patients with mild/moderate and severe/critical illness within different suPAR level intervals. Patients were stratified into three suPAR level groups according to manufacturer's recommendations. A χ^2^ test revealed asymmetric distribution of patients with different disease severity. suPAR, soluble urokinase plasminogen activator receptor.

Based on previous research (47) and guidelines from the manufacturer of the suPAR ELISA, a suPAR of <4 ng/mL supports patient discharge whereas >6 ng/mL supports hospitalisation. Hence, our patients were stratified into three groups based on suPAR levels: low (<4 ng/m), medium (4–6 ng/mL) and high (>6 ng/mL). The frequency of patients with mild/moderate and severe/critical illness was significantly different between the suPAR stratification groups (*P* = 0.004) with none of the severely/critically ill patients having low suPAR levels (Figure 5). Furthermore, a higher frequency of severely/critically ill patients was found in the group of high suPAR compared to mild/moderately ill (Figure 5). The two deceased patients had a baseline suPAR within the group of medium and high suPAR levels, respectively.

## Discussion

The present study confirms but also contributes with new knowledge of suPAR as an independent predictor of organ damage, in this case pneumonia with respiratory failure, and length of hospital stay for patients with different severity of COVID-19 in a Swedish setting. suPAR was significantly elevated at inclusion in patients who later developed severe or critical illness with increased oxygen demand and subsequently had a longer stay at the hospital. The length of hospital stay in this cohort of COVID-19 patients was mainly determined by the need for oxygen supplementation with gradual phasing out and not the need for rehabilitation, since rehabilitation primarily was carried out at another department. Our results are consistent with previous studies (16, 47), but to our knowledge, suPAR has previously not been shown to independently reflect the cohesive length of hospital stay.

Nevertheless, suPAR has previously been shown to correlate with a prolonged stay at hospital due to other serious conditions with excessive inflammation, such as cardiac surgery, pneumonia in children, and burn injuries (48–50). Besides this, the significance of elevated suPAR levels in viral infections such as human immunodeficiency virus, hepatitis B virus, hepatitis C virus, and hantavirus, has been established before the SARS-CoV-2 pandemic. Like its importance in COVID-19, elevated suPAR was demonstrated to significantly correlate with severity and mortality in these conditions (51–55).

The concentration of suPAR in sera correlated with the degree of COVID-19 severity, which has been shown previously (47). In SARS-CoV-2 infected individuals with low admission levels of suPAR (<4 ng/ml), the risk of needing mechanical ventilation and the 14-day mortality was almost non-existent, while levels between 4–6 ng/ml and especially >6 ng/ml were associated with a significantly increased risk. Similar outcomes of stratification of suPAR levels in relation to COVID-19 disease classification were obtained in this study. Interestingly, the cut-off level for severe/critical disease obtained from our study population (5.9 ng/mL) was very close to the manufacturer's cut-off level for the recommendation of hospitalisation (6 ng/mL).

The study cohort reflects the well described characteristics of hospitalised patients with COVID-19, with a male dominance, middle-aged and older individuals, presence of co-morbidities such as CVD, chronic pulmonary disease, obesity, and diabetes (56–58). Although these conditions are known risk factors for severe COVID-19, somewhat surprisingly, we did not find a significant association of co-morbidities, besides obesity, with disease severity. This is partly explained by the limited number of patients in each group. Approximately one tenth of the cohort had an immunosuppressive disorder, whereof two patients became critically ill and one with a newly diagnosed haematological malignancy deceased during the hospital stay. These findings are consistent with prior observations indicating a relatively low risk, compared to the general population, of severe COVID-19 due to immunosuppression (59–63). However, since not all patients with COVID-19 at the hospital were included in this study, definite conclusions cannot be drawn from the proportion of immunosuppressed relative to immunocompetent patients in the cohort. Most of the patients were classified as moderately to severely ill, *i.e.*, needed non-invasive ventilation support mainly in the form of HFNOT. All of them received dexamethasone to reduce the inflammation in the lungs. Sixty percent of the patients were already prescribed corticosteroids at inclusion, which might have influenced the suPAR levels. In fact, corticosteroids have a suppressive effect on suPAR (21, 64). However, among patients in this study, suPAR levels were higher in corticosteroid treated patients, probably indicating dexamethasone treatment as a surrogate marker of severe disease. Similarly, suPAR levels were higher in patients who received remdesivir at inclusion, reflecting severe inflammation and lung involvement of COVID-19 in these patients already early in the course of the disease. Remdesivir treatment was, however, not associated with disease severity, but rather with length of hospital stay. A similar association was in fact made in studies by Anderson et al. (65) and Spinner et al. (66), in which length of hospital stay was shown to be affected by remdesivir treatment and a peak in discharge rates was observed upon completion of the intravenous therapy, suggesting that physicians actually delayed discharge to complete treatment. The small cohort in this study does not allow any definite conclusions to be drawn regarding possible beneficial effects of remdesivir on disease severity.

The main strengths of the study were the prospective design, the well-characterised patient cohort, and that the assessment of clinical variables and review of medical records in all patients was done by one, experienced infectious disease specialist (J.S.). However, some limitations deserve to be mentioned. The range of symptom duration was wide, explained by the fact that some patients were initially receiving care at another pandemic department at the hospital and were not included until they were transferred to the Department of Infectious Diseases. Many elderly patients were excluded because of acute or chronic cognitive impairment, which resulted in a relatively limited study population. For practical reasons, only Swedish and English-speaking patients were included, which might have ruled out patients known to have an increased risk of developing severe COVID-19 (67, 68).

## Conclusions

We show that suPAR is an independent predictor for the development of severe COVID-19 in patients in need of hospital care and supplemental oxygen therapy, and it helps to predict the length of hospital stay and thereby provide support for prioritisation of the care level before the patient's condition deteriorates. Studies on suPAR kinetics during hospital stay and the effect of immunosuppressive therapies on suPAR levels may further clarify the significance of this biomarker in hospitalised patients with SARS-CoV-2 infection.

## Data Availability Statement

The original contributions presented in the study are included in the article/supplementary material, further inquiries can be directed to the corresponding author.

## Ethics Statement

The studies involving human participants were reviewed and approved by The Swedish Ethical Review Authority (Decision number 2020–02580). The patients/participants provided their written informed consent to participate in this study.

## Author Contributions

HE, CI, AG, ML, ÅN-A, and JS participated in conception and design of the study. MG and FH processed serum samples. HE, CI, AG, and JS took part in acquisition, analysis or interpretation of the data. HE, CI, AG, MG, FH, ML, ÅN-A, and JS were involved in writing and critiquing of drafts of the manuscript. HE, CI, AG, MG, FH, ML, ÅN-A, and JS approved the final manuscript for submission. All authors contributed to the article and approved the submitted version.

## Funding

This study was supported by Region Östergötland (Stiftelseförvaltningen, RÖ-931742 (HE); ALF Grant, RÖ-935411 (JS); Regional ALF Grant 2021 (ÅN-A and JS), Vrinnevi Hospital in Norrköping) and SciLifeLab/KAW COVID-19 Research Program (ML).

## Conflict of Interest

The authors declare that the research was conducted in the absence of any commercial or financial relationships that could be construed as a potential conflict of interest.

## Publisher's Note

All claims expressed in this article are solely those of the authors and do not necessarily represent those of their affiliated organizations, or those of the publisher, the editors and the reviewers. Any product that may be evaluated in this article, or claim that may be made by its manufacturer, is not guaranteed or endorsed by the publisher.

## Abbreviations

suPAR: soluble urokinase plasminogen activator
NMR: neutrophil-to-monocyte ratio
NLR: neutrophil-to-lymphocyte ratio
LDH: lactate dehydrogenase
CRF: chronic renal failure
CRP: C-reactive protein
BMI: body mass index
CVD: cardiovascular disease
HFNOT: high-flow nasal oxygen therapy
CPAP: continuous positive airway pressure
eGFR: estimated glomerular filtration rate.
